# Application of Machine Vision System in Food Detection

**DOI:** 10.3389/fnut.2022.888245

**Published:** 2022-05-11

**Authors:** Zhifei Xiao, Jilai Wang, Lu Han, Shubiao Guo, Qinghao Cui

**Affiliations:** Key Laboratory of High Efficiency and Clean Mechanical Manufacture of Ministry of Education, School of Mechanical Engineering, National Demonstration Center for Experimental Mechanical Engineering Education, Shandong University, Jinan, China

**Keywords:** machine vision system, deep learning, food detection, food classification, food Image analysis

## Abstract

Food processing technology is an important part of modern life globally and will undoubtedly play an increasingly significant role in future development of industry. Food quality and safety are societal concerns, and food health is one of the most important aspects of food processing. However, ensuring food quality and safety is a complex process that necessitates huge investments in labor. Currently, machine vision system based image analysis is widely used in the food industry to monitor food quality, greatly assisting researchers and industry in improving food inspection efficiency. Meanwhile, the use of deep learning in machine vision has significantly improved food identification intelligence. This paper reviews the application of machine vision in food detection from the hardware and software of machine vision systems, introduces the current state of research on various forms of machine vision, and provides an outlook on the challenges that machine vision system faces.

## Introduction

The food industry is becoming more competitive and dynamic, and consumers’ awareness of what they are eating is increasing (1). Consumers’ desires for diversified functions of food have gradually increased over the last few years (2). This is due to new demands not only for food’s nutritional needs but also for its health and quality (3). In order to improve the efficiency and quality of food production, efficient and advanced food processing detection methods need to be developed (4).

Food quality and safety are the foundations of food processing and human health, thus classification and inspection of food are critical in the food processing (5). Traditionally, the quality of ingredients was mainly determined by human sensory testing, which is inefficient and subjective (6). The machine vision system can capture various information of food including size and dimensions, appearance and shape, and surface color, etc. It can accurately capture the detailed information of food products, improve the accuracy of monitoring and monitor food processing with limited human errors (7). Because of the advancement of computer technology, machine vision system has been applied in industrial applications, including the food processing industry (8).

**FIGURE 1:**
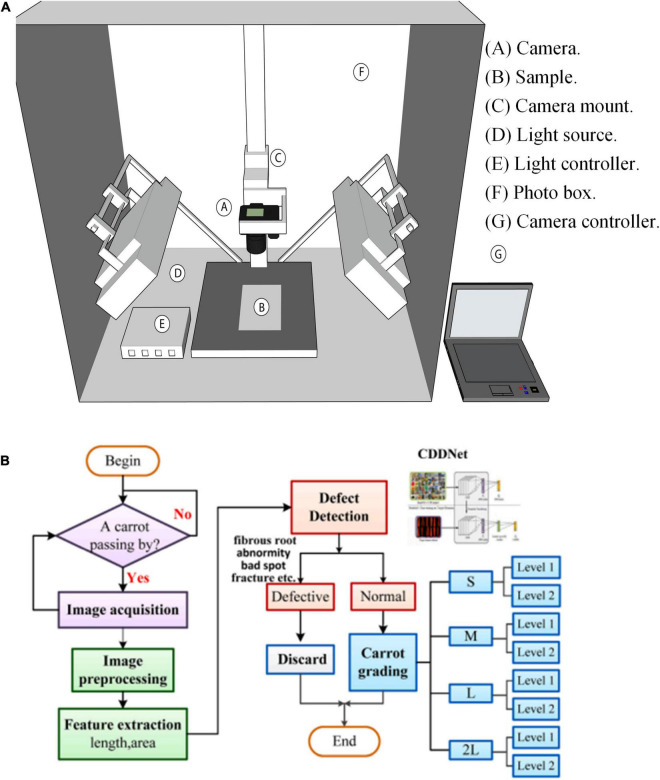
Computer technology applied to food engineering, **(A)** Computer vision systems; **(B)** overall flowchart of deep learning (13).

The main function of computer vision is to simulate the video and graphic information seen by human eyes and to monitor and process existing data image information, which can facilitate technicians to quickly capture sensitive detection indicators in the process of data entry, information integration, data analysis and data labeling (9). Machine vision systems usually include two parts: image information capture and image information

sprocessing. Image information capture is mainly through a variety of hardware devices for real-time acquisition of food image information. Information processing is through the setting of scientific operating procedures, the core stored information for secondary evaluation, to facilitate the technical staff to timely probe, receive, and understand the actual state of low level visual inspection information (10). computer technology is primarily used for food material classification and defect analysis (11). Through machine vision and deep learning, computer technology provides an accurate, efficient, and non-destructive method for detecting and grading agricultural products (12). The computer vision system and deep learning framework are shown in Figure 1.

The purpose of this review is to review the impact of computer technology on food detection and assortment, and to provide a reliable reference for further research on the intelligence and efficiency of the food industry.

## Collection of Tested Food Information

Machine vision system consists of an image acquisition equipment and an image information processing system. Among them, the image acquisition equipment has a variety of acquisition forms, including X-ray, thermal imaging, remote sensing imaging, magnetic resonance imaging, and other acquisition equipment in addition to the common high definition camera (14). Image information processing is the analysis of the acquired food information, mainly extracting and identifying the acquired image information. This process of image acquisition can be seen as the acquisition of image signals and does not involve changes in the interpretation of the image content or meaning (15).

### High Definition Camera Acquisition Equipment

Machine vision for recognizing food information is becoming increasingly popular, such as food defect recognition and ripeness analysis (16). For food defect detection, Patel et al. (17) developed a method using monochrome cameras for automatic mango sorting, and result showed that the system’s detection efficiency and accuracy reached 97.88% and 88.75%, respectively. Noor Fatima et al. (18) used industrial cameras combined with deep learning to develop a device for tracking the adulteration of papaya seeds in black pepper. Furthermore, machine vision can identify and classify food products based on their color. Burce Atac et al. (19) proposed a method to extract average or characteristic color information from digital images of food products, and discussed specific applications for different food products. Ayustaningwarno et al. (20) used computer image analysis to quantify the color distribution of fried mangoes. To verify the efficiency of machine vision, Fitriyono Ayustaningwarno et al. (21) experimentally compared the ability of sensory testing, Hunterlab colorimeter, a commercial machine vision system (IRIS-Alphasoft), and the custom-made machine vision system (Canon) techniques to discriminate among nine vacuum-fried fruit samples.

With the increasingly widespread use of machine vision, it becomes increasingly common to use stereo systems to collect depth information about the environment for object recognition and environmental modeling. To increase the accuracy and authenticity of image information collection, 3D stereo cameras are used in food inspection (22). Stereo camera prototypes are used to capture pairs of images for estimating the size of target foods (23). Su et al. (24) used a depth camera based on machine vision technique to successfully assess the sample quality in 3D space of potatoes. Sepehr Makhsous et al. (25) stated that 3D detection system can monitor food volume for accurate monitoring of diabetic patients’ dietary intake. Yao et al. (26) proposed a potato volume measurement method based on RGB-D camera to achieve automatic monitoring of potato volume. The prediction errors were found to be 9% for regular potatoes and 30% for irregular potatoes through experiments.

Due to the diversity and complexity of the food industrial identification objectives, high definition camera has a number of problems in its application (27). Firstly, high definition camera requires high working conditions and cannot handle image information with high noise and low brightness. In addition, when the quality characteristics are mainly determined by the inherent properties of the sample (composition and internal physical properties), computer vision techniques appear less effective because they are not easily detected from the surface. Furthermore, high definition cameras has limitations in recognizing defects with high color similarity. To improve the recognition ability of machine vision, some corresponding acquisition devices have been developed for the application in food inspection.

### Other Information Collection Equipments

Due to the development and maturity of imaging spectroscopy technology, hyperspectral imaging sensors are used for the accurate detection of food appearance (28). Xi et al. (29) investigated the effect of different peel colors on soluble solids content using hyperspectral imaging and developed a prediction model for soluble solids content. To improve identification efficiency, Li et al. (17) developed an improved watershed segmentation algorithm with morphological gradient reconstruction and marker extraction, which is different from the traditional segmentation algorithm, and applied it to analyze multispectral images. The test results showed that the recognition accuracy of the algorithm was found to be 100%. Zhang et al. (30) developed a multispectral image classification algorithm for the detection of common citrus defects based on visible-NIR hyperspectral imaging technology, and successfully applied it to the detection of citrus defects. For internal food inspection, the information acquisition equipment can use X-ray and magnetic resonance imaging. X-ray devices are used to detect foreign objects inside food by using X-rays through the food surface, and Kazuya Urazoe et al. (31) used X-rays combined with deep learning to successfully apply them to the detection of fish bones.

Magnetic resonance imaging can non-destructively detect and image the structure of food (32). Nakashima (33) developed a set of handheld magnetic resonance sensors consisting of a planar RF coil and a single-sided magnetic circuit for the measurement of the internal fat content of tuna. The temperature of food is one of the key factors affecting the quality of food, and thermal imaging can monitor and control the temperature of food at various points in the process (34). Zeng et al. (35) built a thermal imaging system for pear detection and used an improved deep learning algorithm based on a small sample dataset of thermal images to classify pears with or without bruises. For large-scale agri-food detection, remote sensing technology is used to increase agricultural yields and reduce input losses (36). Romanko and Matthew (37) assessed the effectiveness of remote sensing technology in modeling several important agrochemical parameters, noting that remote sensing technology can promote modern agriculture, improve the efficiency of agrochemical use and reduce environmental pollution. Lin et al. (38) used high resolution (6 m) multispectral satellite imagery SPOT-6 for monitoring powdery mildew in winter wheat in areas with severe disease infection and pointed out the great potential of high resolution multispectral satellite imagery data for crop disease monitoring.

## Image Information Processing Based on Deep Learning

In the food inspection process, image information processing and analysis is at the core of computer vision inspection. The purpose of image processing is to improve picture quality and to address defects such as picked geometric distortion, incorrect focus, picture noise, uneven illumination and camera motion. Image analysis is the process of distinguishing objects (objects to be detected) from the background and generating quantitative information that is used in the subsequent control system for decision making. The processing of image information can be divided into three levels: low level processing (image acquisition and pre-processing), mid-level processing (image segmentation and description) and high level processing (recognition and interpretation).

### Low Level Processing

Computer processing can only process data in digital form, so it is necessary to transform the food information acquired by various collection devices. The relevant information acquired through hardware has certain defects due to the acquisition environment (light, humidity) as well as the equipment (device motion, transmission distance). In order to improve the recognition accuracy of machine vision, pre-processing of the information, including noise reduction, contrast adjustment, etc., is required. Among them, Che et al. (39) performed low order processing of the acquisition information and reflection calibration of the image information in order to reduce the effects of different configurations of camera quantum efficiency and hyperspectral imaging systems, thus improving the subsequent recognition accuracy. Fan et al. (40) corrected the original hyperspectral images using white and dark reference images in order to improve the recognition rate of internal bruises in blueberries, thus eliminating current-induced image noise and errors caused by non-uniform illumination.

### Mid-Level Processing

Intermediate processing focuses on image segmentation, image representation and description. Its main purpose is to select the overall information collected to detect the object region. Image segmentation is one of the most important steps in the whole image processing technique, which can improve the accuracy and recognition efficiency of the subsequent advanced processing. Intermediate level processing has important applications in machine vision. Li et al. (41) proposed an improved watershed segmentation algorithm based on morphological filtering and morphological gradient reconstruction and marker constraints for segmenting rotten points on apples in order to achieve fast automatic recognition of whether a fruit is rotten or not. Che et al. (39) built and compared different bruise extraction models after selecting the region of interest by certain rules. The comparison revealed that the random model algorithm was more suitable than other algorithms for classifying bruises on apples. Luo et al. (42) used an improved watershed segmentation algorithm for bruise detection analysis on multispectral principal component analysis images and obtained an overall detection accuracy of 99.5%. Li et al. (43) used bi-dimensional empirical pattern decomposition for removing noise from multispectral images and further reconstructing the images. And an improved watershed segmentation method with morphological gradient reconstruction, marker extraction and image correction is proposed to segment the decayed regions in fruits using the reconstructed multispectral images.

### High Level Processing (Deep Learning Image Processing)

**FIGURE 2:**
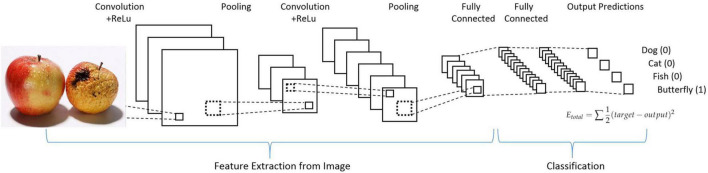
The architecture of a typical CNN.

Common techniques for evaluating food quality include deep learning techniques, statistical learning, fuzzy logic and genetic algorithms. In the last few years, deep learning methods have shown excellent computational performance in several fields, among which the field of computer vision is one of the most prominent application areas of deep learning. Among the various deep learning neural networks, convolutional neural networks have been applied to computer vision, which is particularly suitable for handling various tasks in the field of computer vision, such as image classification, object detection, and semantic segmentation. With the advancement of machine learning, especially the development of convolutional neural networks, the collected food pictures can be further processed to classify the food (44). A CNN model of image recognition is shown in Figure 2. Deep learning automatically extract the features of food shape through neural grid learning, which has the advantage of intelligent recognition compared with traditional image processing methods (45).

Researchers are currently conducting extensive studies on the applications of deep learning in food safety and quality assessment. The recognition steps of conventional deep learning are to collect a large number of required building datasets, then use the datasets to train a network model, and finally use the training model for the detection and recognition of food images (46). Xie et al. (47) used five classical CNN (Densenet-121, ResNet-50, Inception-V3, VGG-16 and VGG-19) models to identify defective carrots and found that CNN models can be an effective method to identify defective carrots through experiments. As an upgrade to machine vision systems, Deng et al. (48) used machine vision combined with deep learning to achieve automatic grading of carrots and improve the intelligence of recognition. Zhu et al. (49) proposed an improved dense capsule network model (Modified-DCNet) for carrot appearance quality detection. This modified model introduces a self-attention layer, which reduces the interference of background information on the recognition task. Moreover, the capsule layer of the modified model uses a locally constrained dynamic routing algorithm to reduce the size of the network parameters and the training load of the network. To improve the accuracy of identification, Rong et al. (50) used deep learning to detect peach varieties and established a VIS-NIR spectral database containing five peach varieties to achieve accurate identification of peach varieties. After testing, it was found that the deep learning-based model achieved the accuracy of 100% in the validation dataset and the accuracy of 94.4% in the test dataset. To improve the recognition efficiency of deep learning, Muoz et al. (51) used different deep learning networks to detect the intramuscular fat content in dry cured ham and found that CNNs can accurately identify and takes less time on the network development by comparison.

Despite its accuracy in image recognition, deep learning has some inherent disadvantages: (1) it requires a large number of food images for input training when performing grid training, (2) deep learning requires high computing performance of the computer, and (3) it takes a lot of time during training.

## Conclusion and Future Perspectives

By summarizing the basic concepts and technologies of machine vision systems, as well as the latest developments and applications of systems in the food detection industry, the following conclusions have been drawn.

(1) The application of computer vision technology in food detection has greatly improved the efficiency of food inspection and promoted the development of food safety, which is an important part of the intelligent process of food engineering. The use of computer vision systems enables automated, objective, rapid and hygienic inspection of a wide range of raw and processed foods.
(2) Machine vision has high recognition accuracy, however, under low light, high humidity, and high noise conditions, there are corresponding detection errors. Therefore, it is necessary to develop high-resolution acquisition devices to improve the quality/accuracy of inspection.
(3) Image processing is considered to be the core of computer vision. With same acquired information, advanced processing software can improve processing efficiency and recognition accuracy. Therefore, it is significant to develop more efficient algorithms to make machine vision more suitable for food inspection.

## Author Contributions

ZX: information collection and writing. JW: figure preparation and proofreading. LH: manuscript framework and writing. SG and QC: writing. All authors contributed to the article and approved the submitted version.

## Conflict of Interest

The authors declare that the research was conducted in the absence of any commercial or financial relationships that could be construed as a potential conflict of interest.

## Publisher’s Note

All claims expressed in this article are solely those of the authors and do not necessarily represent those of their affiliated organizations, or those of the publisher, the editors and the reviewers. Any product that may be evaluated in this article, or claim that may be made by its manufacturer, is not guaranteed or endorsed by the publisher.
